# Autologous Platelet-Rich Plasma for Clitoral Reconstruction: A Case Study

**DOI:** 10.1007/s10508-021-02172-9

**Published:** 2021-11-15

**Authors:** Emily Manin, Gianmarco Taraschi, Sarah Berndt, Begoña Martinez de Tejada, Jasmine Abdulcadir

**Affiliations:** 1 Weill Cornell Medical College, New York, NY USA; 2 Department of Pediatrics, Gynecology, and Obstetrics, Geneva University Hospitals, 30 Blvd de la Cluse 1211, 14 Geneva, Switzerland; 3 Regen Lab SA, En Budron b2, 1052 Le Mont-sur-Lausanne, Switzerland; 4 Faculty of Medicine, University of Geneva, Geneva, Switzerland

**Keywords:** Platelet-rich plasma, Autologous platelet-rich plasma female genital mutilation, Female genital cutting, Clitoral reconstruction

## Abstract

Clitoral reconstruction after female genital mutilation/cutting (FGM/C) is associated with significant post-operative pain and months-long recovery. Autologous platelet-rich plasma (A-PRP) reduces the time of healing and pain in orthopedic and burn patients and could also do so in clitoral reconstruction. In the present case, a 35-year-old Guinean woman who had undergone FGM/C Type IIb presented to our clinic for clitoral reconstruction. Her request was motivated by low sexual satisfaction and body image. We surgically reconstructed the clitoris using the Foldès method and applied plasma and glue of A-PRP. The patient was highly satisfied with the procedure. Two months post-operatively, her pain had ceased entirely and re-epithelialization was complete. We conclude that A-PRP may improve pain and healing after clitoral reconstruction. Extensive studies investigating long-term outcomes are needed.

## Introduction

The World Health Organization (WHO, 2016) defines FGM/C as any procedure involving partial or total removal of the external female genitalia or other injuries to female genital organs for non-medical reasons. Some FGM/C procedures involve the cutting of the glans and the body of the clitoris, but the rest of the tumescent structures (body, crura, bulbs, and corpus spongiosum of the urethra) remain intact (Abdulcadir et al., 2016). The cutting of the clitoris and its dorsal nerve can cause spontaneous or provoked clitorodynia or neuropathic pain due to the growth of post-traumatic neuromas, keloids, and granulomas (Ezebialu et al., 2017). While the exact prevalence of sexual dysfunction and vulvar pain among women with different types of FGM/C is unclear (Sharif Mohamed et al., 2020), scar tissue can restrict the mobility of the clitoral tissue, causing pain or anorgasmia (Ezebialu et al., 2017).

Clitoral reconstruction, the surgical removal of the peri-clitoral scar tissue, and potentially neuromas after FGM/C seem to treat such clitoral pain (Abdulcadir et al., 2017; Foldès et al., 2012). Most patients also seek clitoral reconstruction to “restore” their identity, rid themselves of stigma, or improve their sex lives (Sharif Mohamed et al., 2020). Clitoral reconstruction for women with FGM/C has become increasingly common since it was first described by Thabet and Thabet (2003) and studied on a large scale by Foldès et al. (2012). Since then, seven distinct techniques have been developed to improve outcomes of pain, recovery time, wound dehiscence, hematoma, and esthetic clitoral appearance (Botter et al., 2021; Sharif Mohamed et al., 2020; Wilson & Zaki, 2021). However, due to inconclusive evidence on safety and efficacy, particularly long term, clitoral reconstruction is not yet recommended by any professional organization, including the WHO.

With regard to pain and recovery, most long-term outcomes are reported to only one-year post-operatively and include small samples (Sharif Mohamed et al., 2020). In our experience, post-operative pain is present until re-epithelization of the clitoris is achieved, up to three months post-operatively (Abdulcadir et al., 2015). The source of post-clitoral reconstruction pain is the exposed nerve endings of the de-epithelialized clitoris, which affects the patient’s daily life and may induce relapse of PTSD (Sharif Mohamed et al., 2020). To address this, Mañero and Labanca (2018) grafted mucosal tissue from the posterior vaginal wall onto the neo-clitoris to minimize exposed nerve endings. However, two (6.3%) patients in this study experienced partial necrosis of the vaginal wall graft, and little is known about the graft’s impact on clitoral sensation (Mañero & Labanca, 2018). The present case study aims to improve post-clitoral reconstruction pain and expedite re-epithelialization without compromising clitoral sensitivity using autologous platelet-rich plasma (A-PRP) after clitoral reconstruction according to the Foldès technique, the most well-studied and reproducible clitoral reconstruction method.

A-PRP, plasma with platelet concentrations at least three times above basal levels, is an autologous product made by centrifuging blood from a patient’s vein to concentrate the platelets; the resulting A-PRP is reinjected into the area of interest (Dhillon et al., 2012). It secretes hundreds of factors, most of which promote cell adhesion, collagen synthesis, wound regeneration, and vascularization (Dhillon et al., 2012). A-PRP improves wound healing, revascularization, recovery time, and pain, particularly in the fields of orthopedics and maxillofacial surgery (Dhillon et al., 2012). It is hypothesized to reduce neuropathic pain specifically (Kuffler, 2013).

While not yet approved by the U.S. Food and Drug Administration (FDA), A-PRP is promoted by the International Cellular Medicine Society (2011) for use in musculoskeletal injuries. There is no formal recommendation from obstetrics and gynecology societies, but it is a growing area of research in these disciplines. A non-randomized prospective study of patients suffering from sexual dysfunction, urinary stress incontinence, and overactive bladder reported that peri-clitoral and vaginal A-PRP injections improved symptoms for most patients without severe side effects (Neto, 2017). Another prospective trial of 55 patients undergoing major gynecologic surgery, such as laparoscopic-assisted vaginal hysterectomy, abdominal hysterectomy, and advanced urogynecologic procedures coupled with intraoperative A-PRP injections, found significantly lower pain in the immediate post-operative period compared to a control group (Fanning et al., 2007). A randomized trial of 120 women with benign cervical ectopy found that applying A-PRP twice to the site resulted in significantly faster re-epithelialization than women who received laser treatment (Hua et al., 2012). A-PRP glue, an alternative formulation of PRP that is mixed with a gelation inducer, has been shown to have positive effects on healing in the fields of plastic, orthopedic, and maxillofacial surgery and has slower, longer-acting effects due to platelets being activated in situ. (Hersant et al., 2016). A-PRP has been suggested for use in clitoral reconstruction (Seifeldin, 2018) and to decrease post-operative pain (Botter et al., 2021). Given its potential to expedite healing and mitigate pain, as well as its safety in peri- and intra-clitoral injections, we decided to use liquid A-PRP and A-PRP glue for the present case.

## Case Report

**Fig. 1:**
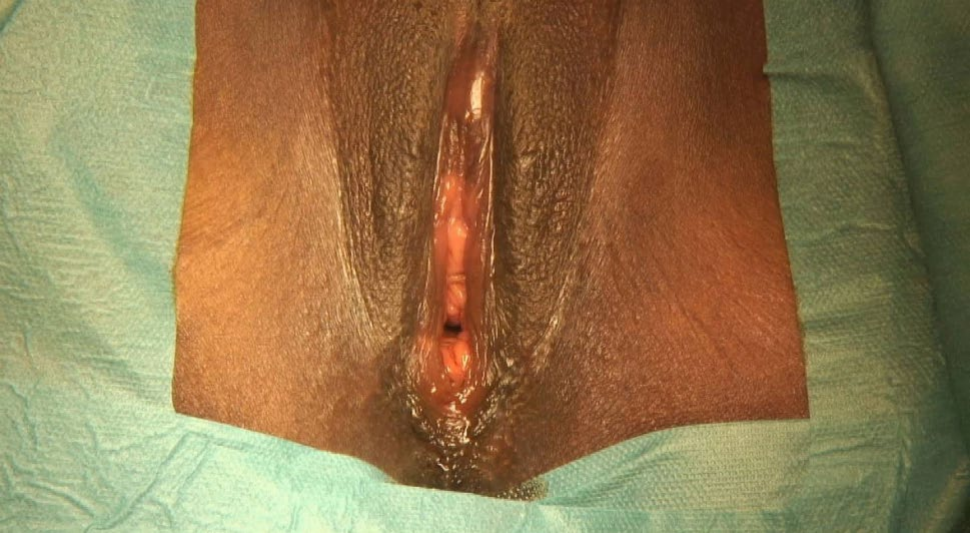
Female genital mutilation/cutting (FGM/C) involving the cutting of the labia minora and the clitoris. Scarring over the clitoris can be observed

A 35-year-old G3P1021 Guinean woman with Type IIb FGM/C who had three pregnancies and one living child was referred to our specialized clinic for women and girls with FGM/C at Geneva University Hospitals for clitoral reconstruction. She requested clitoral reconstruction to improve her body image and sexual response and satisfaction. The patient had no remarkable medical history besides FGM/C, from which there had been no other short- or long-term psychological or physical complications. Her clitoris was palpable, and the scar over it was flexible (Fig. 1).

The patient remembered undergoing FGM/C at the age of 11. She had been living in Switzerland for more than ten years. She was in the process of a divorce from a Western partner who accused her of being unable to enjoy sex and reach orgasm because of the cutting. She was deeply affected by this comment and felt stigmatized and dissatisfied with her sexual experiences and genital appearance. The patient denied dyspareunia and clitoral pain. She was recently sexually active with a new Western partner and could experience orgasm with him and during masturbation.

According to our protocol as well as experts’ opinions and current recommendations for women requesting clitoral reconstruction, our patient received sexual counseling and education on her genital anatomy, physiology, and sexual response as well as 2 months of psychosexual therapy pre-operatively (Sharif Mohamed et al., 2020; World Health Organization, 2016). The patient was counseled on the details of the clitoral reconstruction procedure, including surgical outcomes and risks, and offered an intraoperative clitoral injection of A-PRP and application of A-PRP glue. The patient was informed that A-PRP is experimental in the context of clitoral reconstruction and counseled appropriately on the risks. She provided a written informed consent for the surgery, the intraoperative administration of A-PRP, and the publication of the present case report. Her new partner was supportive of the surgery.

**Fig. 2:**
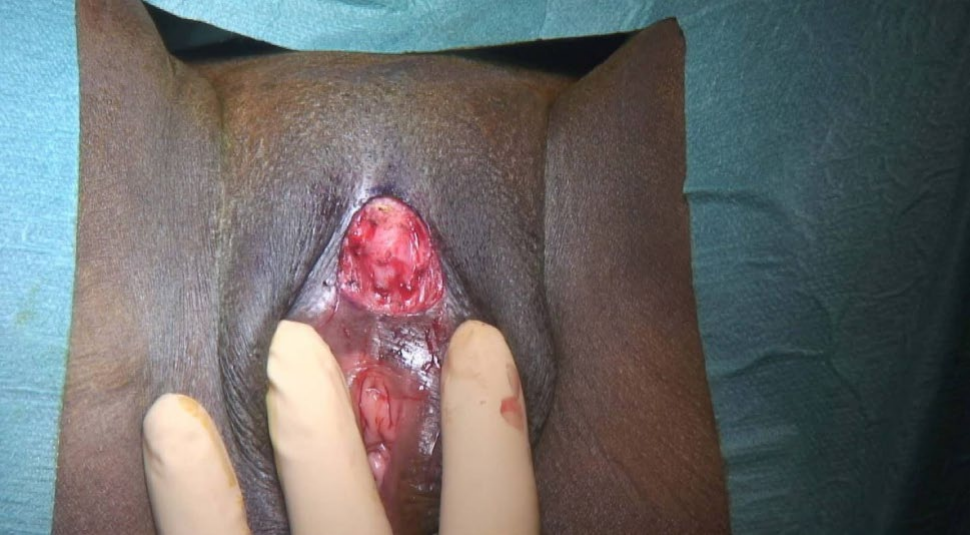
Clitoral tissue is visualized during the clitoral reconstruction procedure, after the excision of the cutaneous scar

**Fig. 3:**
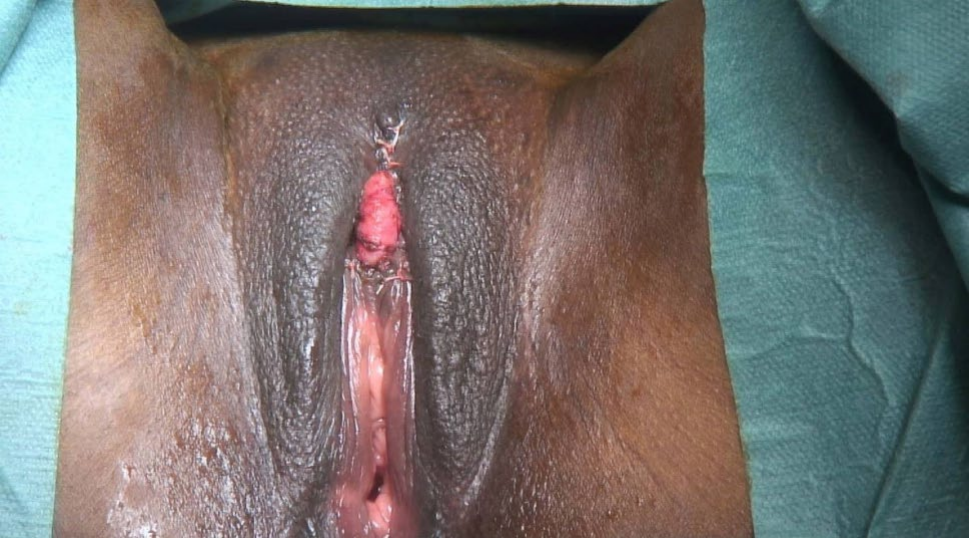
Neo-clitoris is positioned at the level of an unaltered anatomical clitoris

**Fig. 4:**
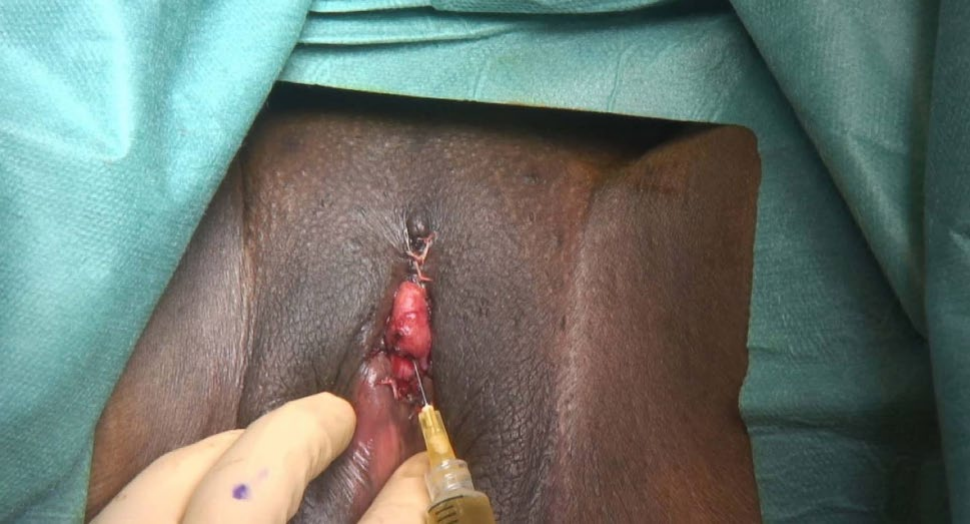
Injection of autologous platelet-rich plasma (A-PRP) into the body of the clitoris

We performed clitoral reconstruction according to the Foldès method under general anesthesia (Figs. 2 and 3) (Foldès et al., 2012). Platelet-rich plasma (PRP) was obtained through centrifugation of blood collected from the patient according to the RegenKit-BCT® procedures (RegenLab SA, Le Mont sur Lausanne, Switzerland). 5.5 ml of A-PRP and 2.2 billion platelets were obtained from 10 ml of blood. Five cubic centimeters of A-PRP plasma was injected at multiple points into and around the neo-glans of the clitoris (Fig. 4). Autologous glue was prepared from the patient’s own blood with RegenKit®-Surgery device and applied peri-operatively above the neo-clitoris. A-PRP glue is similar to A-PRP but is mixed with a gelation inducer just prior to application. There were no intraoperative complications, and the patient left the hospital the same day. We recommended the patient apply aqueous chlorhexidine for the next two days and take acetaminophen, ibuprofen, and tramadol for pain. One week post-operatively, she did not experience any pain with the three aforementioned oral medications. We prescribed 500 mg of metronidazole twice per day for five days and silver sulfadiazine for abundant yellow discharge.

**Fig. 5:**
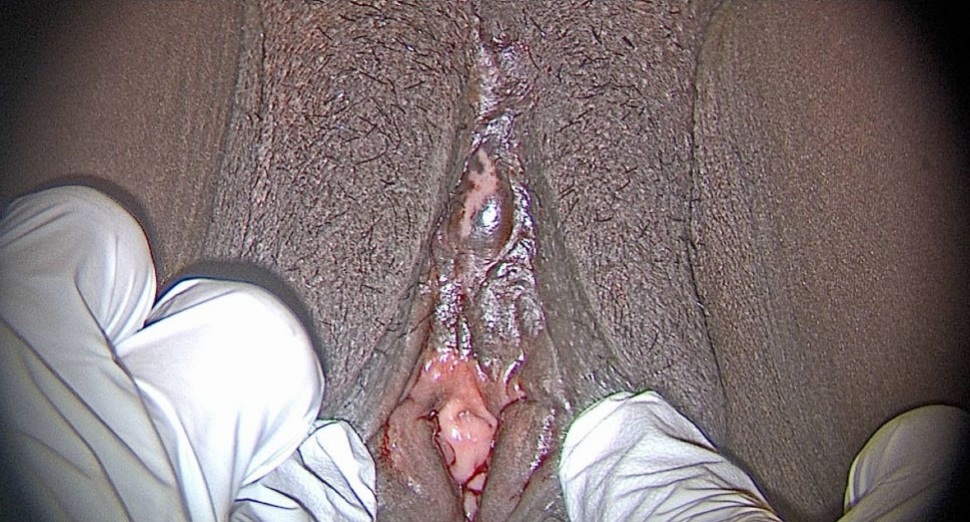
Patient’s vulva and reconstructed clitoris two months post-operatively. Re-epithelialization is nearly complete (this picture was taken, while the patient was menstruating)

At 21 days post-operatively, the patient was only taking ibuprofen three times per day and acetaminophen four times per day. She was delighted with the procedure. Thirty-nine days post-operatively, she was able to wear underwear and no longer had pain. Her wound had nearly re-epithelialized after 60 days (Fig. 5). Seventy days post-operatively, she had returned to work without pain, although she sometimes experienced small electric shocks. She continued to use dexpanthenol ointment two to three times per week. We informed the patient that she might begin sexual intercourse and masturbation 10–11 weeks post-operatively. At six months, she had resumed sex and had no pain. She experienced orgasm and was satisfied with her body image. The patient declared that the most important effect of the surgery was an improvement of her body image.

## Discussion

The present case study describes the first clitoral reconstruction surgery using injection and application of A-PRP-rich plasma and glue to reduce pain and expedite clitoral re-epithelialization. The patient, who had undergone FGM/C Type IIb in Guinea, desired clitoral reconstruction to improve her sexual pleasure and body image. She underwent thorough sexual education and counseling as well as two months of psychosexual therapy pre-operatively. Pre-operative psychosexual care can improve genital self-image and knowledge as well as sexual response. It explores and addresses any unrealistic expectations about the surgery and possible past traumas other than FGM/C that could affect patients’ sexual response. Many women consulting for clitoral reconstruction do not opt for the surgery after such pre-operative care as their body image, identity, and sexuality have significantly improved without clitoral reconstruction (Sharif Mohamed et al., 2020).

While many different variations on Foldes’s clitoral reconstruction technique exist, post-operative pain and recovery time remain major issues. This is particularly significant in a population with a high prevalence of trauma from FGM/C and other sexual or non-sexual traumatic events (Fox & Johnson-Agbakwu, 2020). In the present case, post-operative pain had ceased and re-epithelialization was nearly complete after two months, a seemingly shorter recovery time than that reported for clitoral reconstruction surgeries without PRP as well as in our clinical experience. Our patients who underwent clitoral reconstruction without A-PRP by the same surgeon who performed the present case experienced pain for two to three months until they had fully re-epithelialized (Abdulcadir et al., 2015). A personal communication on a survey of 31 surgeons worldwide who conduct clitoral reconstruction found that three months post-operatively, only 58% of surgeons had pain-free patients (Bah et al., n.d.).

A shortcoming of the present case and associated literature review is that FGM/C research is confounded and limited by the different types of cuttings, sexual history and violence, the complexity of sexuality, small sample sizes, use of non-validated scales to quantify pain, and heterogeneity of study design. There is also no established protocol for the mode, temporality, or quantity regarding A-PRP preparation and administration after clitoral reconstruction (Dhillon et al., 2012). Repeated injections could expedite the healing, as was shown in a meta-analysis of patients with osteoarthritis of the knee and suggested in a prospective study of patients with diabetic foot ulcers (Kontopodis et al., 2016; Vilchez-Cavazos et al., 2019). The maximum number of A-PRP injections of which we are aware was a biweekly regimen for diabetic foot ulcer patients for 16 weeks (or until the ulcer was healed), but no limit is explicitly established in the literature (Kontopodis et al., 2016). In keeping with many studies of gynecologic surgical applications of A-PRP, we opted for a single application of A-PRP (Dawood & Salem, 2018). In the present case, we combined A-PRP injections and autologous glue application on the neo-clitoris to promote direct and long-term regenerative effects. The platelets in the autologous glue were immediately activated by autologous thrombin present in the mixture, while in liquid A-PRP injection, platelets were activated in situ, allowing a slower and longer release of the growth factors. No studies to our knowledge have directly compared the clinical effects of liquid A-PRP and A-PRP glue.

The long-term effects of A-PRP are not well documented; however, to our knowledge, there have been no long-term adverse outcomes, and multiple studies, primarily in the field of orthopedics, have demonstrated shortened recovery time, improved quality of scar tissue, improved functional and mechanical outcomes, and increased vascularization (Dhillon et al., 2012). A study of 91 patients reported that three A-PRP injections for knee osteoarthritis and degenerative cartilage lesions were safe and had lasting benefits at 12 but not at 24 months post-injection (Dhillon et al., 2012). Additionally, a study of 62 patients who underwent three treatments of A-PRP and carbon dioxide laser for stress urinary incontinence (SUI) found significantly improved SUI symptoms at 12 months after the last treatment (Behnia-Willison et al., 2020). However, the long-term impacts of a single PRP injection, particularly in gynecologic disciplines, are yet to be definitively shown (Dawood & Salem, 2018).

PRP injection is not without risk. Risks inherent in any injection include infection, scar tissue formation, and calcification at the injection site (Dhillon et al., 2012). Neurovascular injury could occur, although we took care not to inject the PRP near the dorsal nerve. Two cases reported in the literature described patients developing severe allergic reactions after PRP injections, challenging the common assumption that autologous products are harmless (Kaux et al., 2014; Latalski et al., 2019).

Although it is not yet approved by the FDA, this case indicates that A-PRP may be an effective way to improve pain and re-epithelialization after clitoral reconstruction for patients with FGM/C. More extensive studies evaluating different modes, quantities, and time frames of A-PRP administration are needed before we can definitively tout A-PRP’s utility in clitoral reconstruction. Research to distinguish neuropathic, nociceptive, and inflammatory pain is crucial to target and treat post-clitoral reconstruction pain effectively. Personal communication and experience have shown a general increase in post-operative pain on days five to seven after clitoral reconstruction (Bah et al., n.d.). We hypothesize that this could be due to exposed or damaged nerve endings producing ectopic discharges (Devor & Rappaport, 1990), regenerating nerve sprouts developing into traumatic neuromas (Oliveira et al., 2018), a chemokine increase upregulating AMPA receptors (Wang et al., 2020), and/or proliferation of granulation tissue (Lokmic et al., 2006), but further study is needed.

Future studies should use validated questionnaires pre- and post-operatively to evaluate pain severity and duration, sexual function, sexual satisfaction, and genital self-image. Additional post-operative questionnaires should measure operative recovery time and short- and long-term complications of clitoral reconstruction. Further research should also attempt to control for confounding psychosocial, acculturative, environmental, and biological factors using a control group without clitoral reconstruction.

Studies could also address the following topics: re-epithelialization (and its association with patients’ reported pain), objective measurements of clitoral and vulvar sensitivity, benefits of intraoperative sensory mapping for avoidance of neurological damage during clitoral reconstruction, use of pain medication intra- and post-operatively (e.g., pudendal block), use of topical testosterone, lidocaine and anticonvulsants for post-clitoral reconstruction pain alone or in combination with A-PRP, and esthetic results and patient satisfaction.
